# Stroke Survivors Have Almost Three Times Higher Risk of Depression: A Systematic Review and Meta-Analysis

**DOI:** 10.3390/jcm14238410

**Published:** 2025-11-27

**Authors:** Aryan Naghedi, Raquel Delgado-Mederos, Cristofol Vives-Bauza

**Affiliations:** 1 Neurobiology, Research Unit, Health Research Institute of Balearic Islands (IdISBa), Hospital Universitari Son Espases, 07120 Palma, Spain; a.naghedi@uib.eu (A.N.); raquel.delgado@ssib.es (R.D.-M.); 2 Department of Biology, Institut Universitari d’Investigacions en Ciències de la Salut (IUNICS), University of Balearic Islands (UIB), Crta, Valldemossa, 79, 07010 Palma, Spain; 3 Neurology Service, Neurovascular Area, Hospital Universitari Son Espases (HUSE), 07120 Palma, Spain

**Keywords:** depression, stroke, prevalence, meta-analysis, odds ratio

## Abstract

**Background**: Post-stroke depression (PSD) is one of the most frequent and important complications following stroke that adversely affects conditions such as functional recovery and the patient’s quality of life. Meanwhile, the prevalence proportion of PSD has been widely documented, ranging from 20 to 60%; the relationship between stroke and the manifestation of PSD, quantified with the odds ratio (OR), has been less explored. The primary aim of this meta-analysis is to determine the prevalence OR of suffering depression in stroke survivors. The prevalence proportion of PSD was also analyzed as a secondary aim. **Methods**: A pre-registered meta-analysis designed based on PRISMA guidelines with searches from inception to 23 September 2024 was carried out on PubMed, Web of Science, and SCOPUS databases. Studies reporting the prevalence OR associated with PSD manifestation were eligible for inclusion to achieve the primary aim. Twenty-six comparative studies, including a total population of 947,853 people, met the inclusion criteria. PSD prevalence proportion was extracted from 245 articles, including 493,681 stroke patients. Quality assessments were performed using the Newcastle–Ottawa Scale (NOS). Data were meta-analyzed using a random-effects model. **Results**: Compared with the control population, stroke survivors had higher odds of developing PSD (OR: 2.71; 95% CI: [2.29–3.22]). Prevalence of PSD was 34.46 ± 16.48. **Conclusions**: Stroke survivors have almost 3 times higher probability of suffering depression after stroke than the general population, and almost one third of stroke patients will suffer PSD.

## 1. Introduction

Stroke is characterized by an interruption of blood flow to critical regions of the brain, leading to irreversible damage and long-term effects on the nervous system. The underlying etiology of stroke can be thrombotic, hemorrhagic, or embolic, with most cases (around 85%) being ischemic in nature. Stroke is the second leading cause of death and the third leading cause of acquired disability in adults worldwide [1,2,3,4].

One of the major contributors to stroke-related disability is associated with the development of neuropsychiatric disorders. Post-stroke emotional and mood disorders include depression, anxiety, emotional incontinence, anger propensity, and fatigue [5]. These emotional disturbances negatively impact clinical outcomes for patients [6].

Depression worsens functional recovery after stroke, reduces quality of life, leads to less efficient use of rehabilitation services, and increases mortality [7]. Longitudinal studies have shown that post-stroke depression (PSD) increases its prevalence during the first few weeks following the stroke and seriously impairs cognitive recovery. Interestingly, more than half (53%) of patients who suffer from early-onset PSD (within the first 3 months after the stroke) end up with persistent depression [8,9,10].

The estimated prevalence of PSD is around 30–35%, with reported rates ranging from 20 to 60% [11,12]. Several observational studies and systematic reviews have reported prevalence proportions of PSD in varying degrees and formats. However, despite the abundance of primary studies, it is surprising that no systematic review focusing specifically on comparative studies with a control arm is currently available [13].

The OR, derived from longitudinal, case–control, and cross-sectional studies, offers a more robust measure of association than simple percentages. It accounts for the relative likelihood of an outcome following a specific exposure, thereby providing deeper insight into potential individualized risk. For this reason, we aimed to conduct a meta-analysis of all peer-reviewed, two-armed primary studies to calculate a pooled OR for PSD [14].

## 2. Methods

### 2.1. Study Design and Search Strategy

A pre-registered systematic review and meta-analysis (INPLASY registration number: INPLASY202440106) was conducted on studies reporting the prevalence of depression as a consequence of brain stroke published from inception up to 23 September 2024. The search was performed across major medical databases, PubMed, Web of Science, and SCOPUS, in accordance with the PRISMA 2020 guidelines [15].

A comprehensive and unrestricted search and inclusion/exclusion protocol was developed based on the following PECO framework: Patients: adult patients diagnosed with stroke; Exposure: brain stroke, irrespective of type, severity, or setting; Comparison: non-stroke control group; Outcome: odds of depression following stroke.

The PubMed query was *((((stroke[Text Word]) OR (post-stroke[Text Word])) OR (stroke[MeSH Terms])) AND ((depression[Text Word]) OR (depression[MeSH Terms]))) AND ((((prevalence[Text Word]) OR (prevalence[MeSH Terms])) OR (incidence[Text Word])) OR (incidence[MeSH Terms]))*. Accordingly, *stroke OR post-stroke (Topic) and depression (Topic) and incidence OR prevalence (Topic)* and *TITLE-ABS-KEY ((stroke OR post-stroke OR poststroke) AND depression AND (prevalence OR incidence))* queries were applied in Web of Science and SCOPUS databases, respectively.

### 2.2. Inclusion and Exclusion Criteria

The inclusion screening process consisted of two main stages: (1) title and abstract screening; and (2) full-text screening. During the title and abstract screening, studies that clearly did not report on PSD were excluded. In the full-text screening stage, studies were categorized into three groups: 1. Excluded: Studies that did not report the prevalence of PSD; 2. Included for pooled prevalence analysis: Studies that reported the prevalence of PSD only as a percentage. For these, only the sample size and percentage of stroke survivors with depression were recorded; 3. Included for pooled OR analysis: Comparative studies with a control group that reported our outcome of interest (prevalence OR with 95% confidence intervals (CIs)) and prevalence proportion of PSD. These studies underwent complete data extraction and quality assessment.

### 2.3. Data Extraction and Quality Assessment

In this systematic review, the prevalence OR was set as the main outcome of interest hence, all studies reporting OR or sufficient data to calculate the OR with a 95% CI [95% CI], were chosen for quality assessment and complete data extraction, consisting of first author’s name, publication year, sample size, age, sex, country where the study was conducted, follow-up duration and the scale implemented for depression assessment.

#### 2.3.1. Risk of Bias Assessment

As recommended, for quality or risk of bias assessment of cohort and case–control studies, the Newcastle–Ottawa Scale (NOS) was applied [16]. Also, to evaluate cross-sectional studies, an adapted version of NOS was used, which is provided in Supplementary Materials. The studies were divided based on their risk of bias as: scores 0 to 3 (high risk of bias), 4 to 6 (average risk of bias), and 7 to 9 (low risk of bias).

#### 2.3.2. Assessment of Evidence Certainty

The GRADE (Grading of Recommendations Assessment) approach was applied to evaluate the certainty of the evidence [17,18,19]. As all included studies were observational in design, the initial GRADE level was classified as low-quality evidence. The certainty of each study was subsequently downgraded in the presence of risk of bias, inconsistency, indirectness, imprecision, or potential publication bias, and upgraded when a large magnitude of effect, a dose–response relationship, or plausible confounding was identified.

The detailed workflow used to assess the included studies according to the GRADE framework is presented in Table 1. In this evaluation, the odds of post-stroke depression were considered the primary outcome of interest, and all criteria for upgrading or downgrading the certainty of the evidence were defined in relation to this research question. This structured approach ensured that judgments regarding evidence quality were directly aligned with the clinical relevance and methodological context of the included studies.

During both screening processes for inclusion and the quality assessment stages, two authors (AN and RD) performed separate screening processes, and disagreements were solved by consulting the third author (CV).

EndNote 21 and Microsoft Excel 2024 software were used for the above-mentioned data extraction and quality assessment process.

### 2.4. Statistical Analysis

In this meta-analysis, the OR with [95% CI] was the primary outcome of interest. The effect size was expressed as the log OR with its standard error. Owing to heterogeneity among the included studies, a random-effects model was used to calculate the pooled estimate, presented in a forest plot.

Publication bias was assessed qualitatively using Begg’s funnel plot and quantitatively using Begg and Mazumdar’s rank correlation and Egger’s regression tests. When discrepancies arose between these methods, Duval and Tweedie’s trim-and-fill analysis was also reported [20,21].

Heterogeneity was assessed using the I^2^ and Q statistics, along with their corresponding *p*-values. To evaluate the sensitivity of the meta-analysis results to individual studies, a one-study-removed sensitivity analysis was performed and presented as a forest plot. In this analysis, each primary study was sequentially excluded, and the overall effect size was recalculated to assess the robustness of the findings.

All analyses were conducted using Comprehensive Meta-Analysis software (Version 4, Borenstein, Hedges, & Rothstein, 2022). Statistical significance was set at *p* < 0.05.

IBM SPSS Statistics (version 27) was applied to calculate the mean ± SD of PSD prevalence proportion and to generate the associated distribution chart.

## 3. Results

### 3.1. Search Results

**Figure 1:**
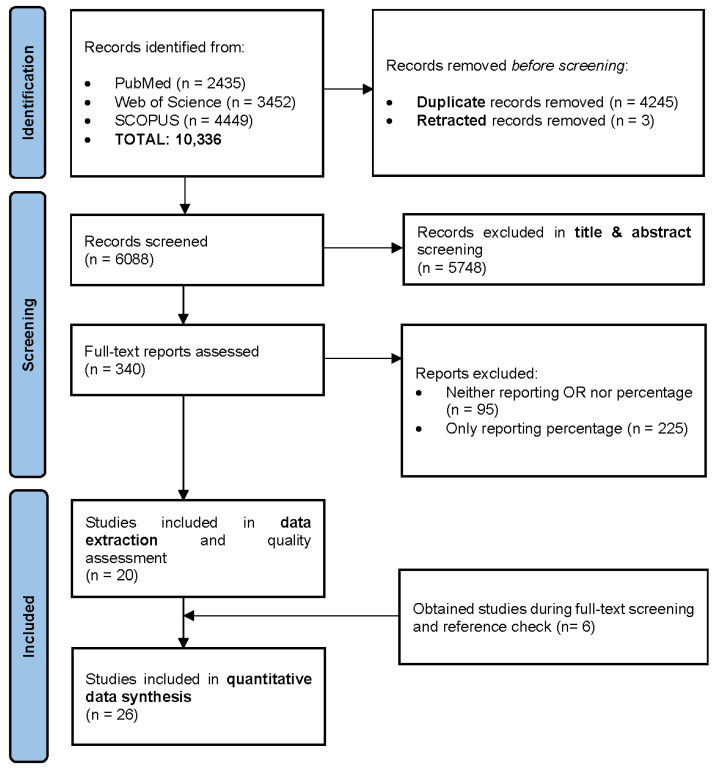
PRISMA flow diagram.

The initial search retrieved a total of 10,336 published papers. After removing 4245 duplicates and 3 retracted articles, 6088 studies were screened based on their titles and abstracts. Of these, 5748 papers were excluded, and 340 studies were selected for full-text screening, as illustrated in Figure 1.

During the full-text screening, 95 articles were excluded. A total of 225 primary studies reporting the prevalence proportion (%) of PSD were separately recorded for prevalence estimation. Additionally, 20 comparative studies with control arms, reporting both prevalence OR and the prevalence proportion of depression following stroke, were finally enrolled for complete data extraction and quality assessment.

**Figure 2:**
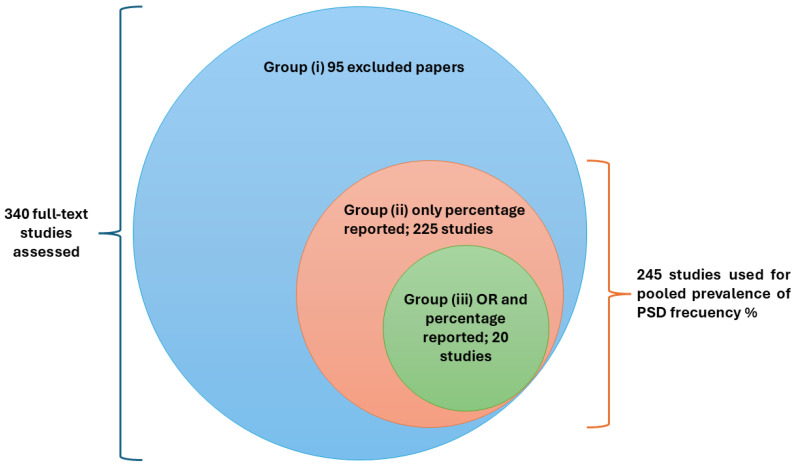
Study groups.

These study groups are summarized in Figure 2.

During the full-text screening phase, six aging cohort studies were also identified and directly included in the data synthesis [22,23,24,25,26,27]. These cohorts had previously been analyzed in a similar meta-analysis with a different study design [28].

### 3.2. Studies and Patients’ Characteristics

This meta-analysis encompasses a total number of 947,853 participants, comprising 349,243 individuals with brain stroke and 598,610 controls. The reported OR [95% CI] ranged from 0.90 [0.42–1.92] to 42.68 [2.50–727.47].

Among the 26 included studies, the earliest was published in 1994 and the most recent in 2024, spanning three decades of medical literature. The primary studies varied widely in the depression assessment tools used, and the follow-up periods ranged from no follow-up to as long as 10 years.

Notably, Brodaty et al. reported their findings at two different follow-up intervals, 3 months and 15 months, which led to the inclusion of 27 data points derived from 26 studies.

The study designs included longitudinal, case–control, cross-sectional, and retrospective cohort studies. Detailed information is presented in Data Extraction Table 2. Of all the included studies, four did not report a statistically significant increase in the prevalence of depression among stroke survivors compared to control groups; the corresponding OR values in these cases are highlighted in red in the data extraction table [25,29,30,31].

### 3.3. Quality Assessment

The results of quality assessment are presented in Table 3. The overall quality of the published evidence was acceptable, and importantly, no studies with a high risk of bias were included. The overall certainty of the evidence was predominantly moderate, with individual studies ranging from very low to high.

It is also worth noting that the six aging cohorts directly included in the quality assessment and data synthesis phase were categorized as having an average risk of bias, each receiving a score of 6 out of 9. All these cohorts were downgraded one level in the GRADE scoring system for indirectness, as their populations were not specifically intended to study stroke patients. This downgrade primarily reflects the fact that their research focus was not on depression, and the cohort arms were not adjusted accordingly. Therefore, this score should not be interpreted as a criticism of the strong methodology of these cohorts, but rather as an indication of the lower relevance of their data to our specific review question.

### 3.4. Meta-Analysis Results and Frequency Analysis

**Figure 3:**
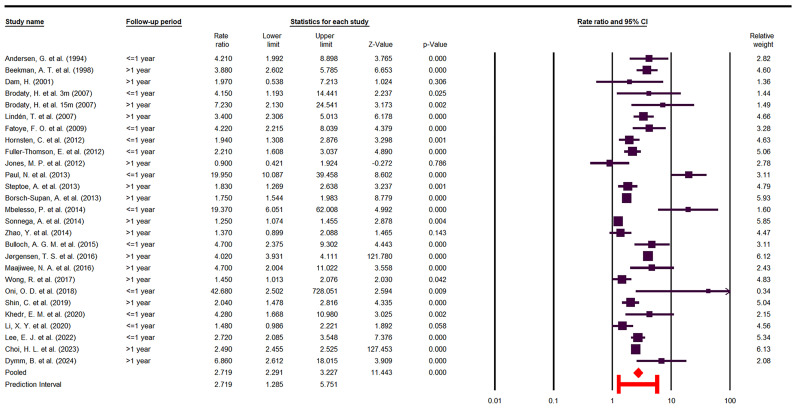
Forest Plot odds ratio (OR) [10,22,23,24,25,26,27,29,30,31,32,33,34,35,36,37,38,39,40,41,42,43,44,45,46,47].

The present study reveals the concerning finding that stroke survivors have nearly a threefold higher risk of depression compared to the general population (OR = 2.71 [2.29–3.22]), as shown in the forest plot (Figure 3).

As expected, there is substantial heterogeneity among the included studies (I^2^ = 98.32, Cochrane Q = 1551.52, *p* < 0.05), which persisted across various subgroup analyses. This suggests that the high variability arises from the heterogeneous nature of the primary studies.

**Figure 4:**
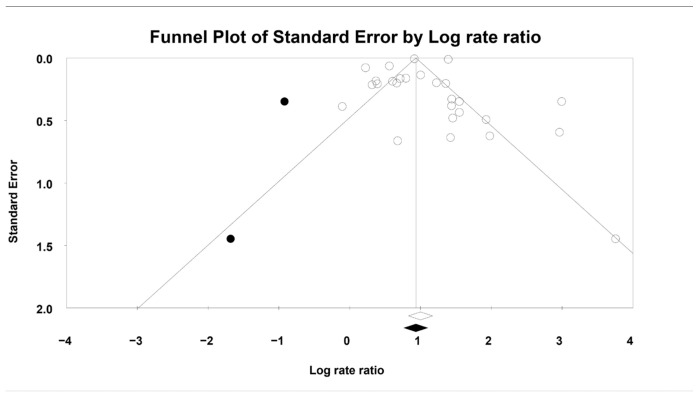
Publication bias. The black dots represent hypothetical studies imputed to counterbalance the potential publication bias. Real studies are shown as white circles. Black diamond indicates the pooled effect, including imputed studies. White diamond reflects the actual pooled effect.

Assessment of publication bias using Begg’s funnel plot suggested asymmetry, indicating that two studies might have contributed to potential bias (Figure 4, displaying both observed and imputed studies). The quantitative analyses yielded partially inconsistent results: Egger’s regression test showed no evidence of publication bias (*p* = 0.94), whereas Begg and Mazumdar’s rank correlation indicated possible bias (*p* < 0.05, Kendall’s tau = 0.35). Therefore, the Duval and Tweedie trim-and-fill test method was applied. After imputing the two hypothetical studies identified in the funnel plot, the pooled OR [95% CI] changed minimally, from 2.71 [2.29–3.22] to 2.54 [2.14–3.01], suggesting that the robustness of the findings remained intact.

**Figure 5:**
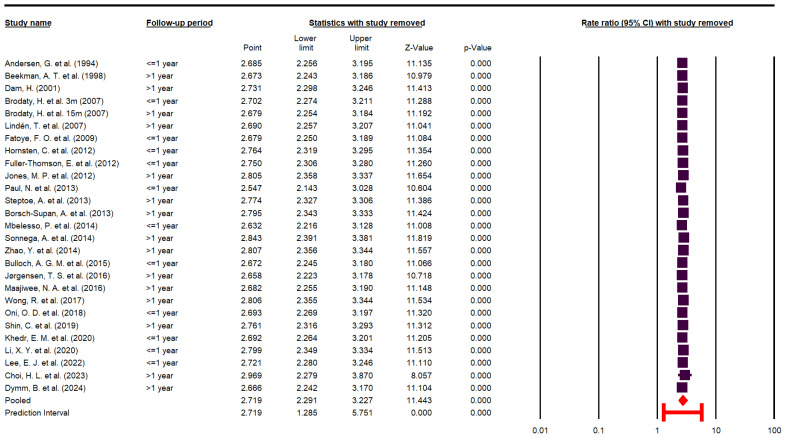
Sensitivity analysis [10,22,23,24,25,26,27,29,30,31,32,33,34,35,36,37,38,39,40,41,42,43,44,45,46,47].

To evaluate the influence of individual studies on the pooled effect, a leave-one-out sensitivity analysis was performed. As illustrated in Figure 5, the exclusion of any single study did not materially alter the overall effect estimate, indicating that no individual study exerted undue influence on the pooled results.

**Figure 6:**
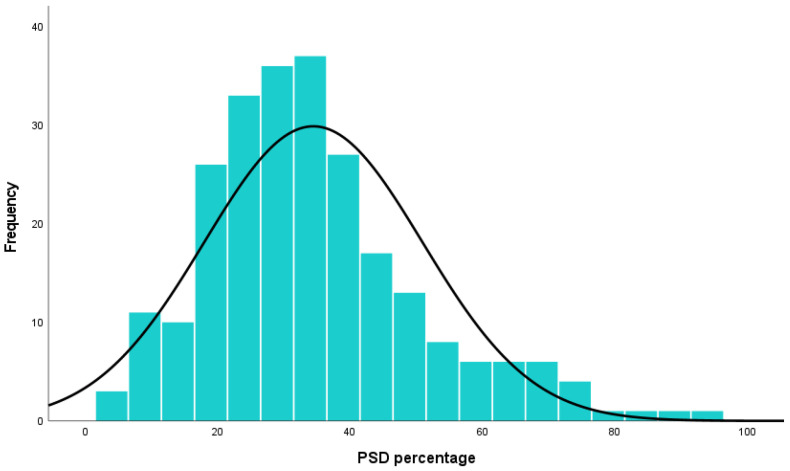
Post-stroke depression percentage (%).

In addition, the mean prevalence of PSD was calculated as 34.46% ± 16.48, based on 245 studies encompassing 493,681 stroke survivors. These findings are presented in the forest plot shown in Figure 6.

### 3.5. Subgroup Analysis

To better understand the course of depression in stroke survivors, studies were stratified by follow-up duration into two groups: ≤1 year and >1 year. This subgroup analysis did not reduce heterogeneity within either group: I^2^ = 83.58, Cochrane Q = 67.02, *p* < 0.05 for the ≤1-year group; and I^2^ = 99.05, Cochrane Q = 1484.49, *p* < 0.05 for the >1-year group.

**Figure 7:**
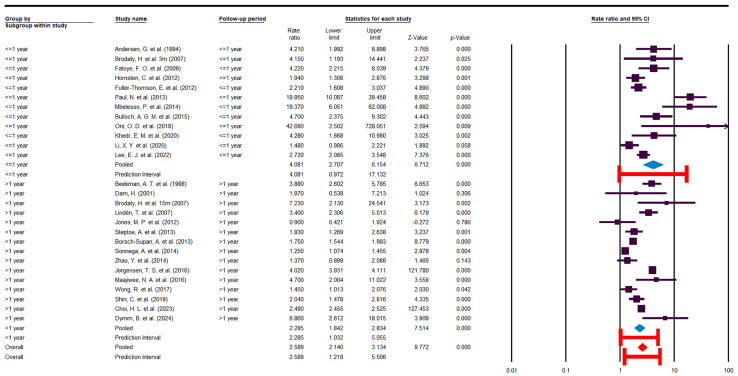
Subgroup analysis [10,22,23,24,25,26,27,29,30,31,32,33,34,35,36,37,38,39,40,41,42,43,44,45,46,47].

As shown in Figure 7, the risk of depression during the first year after stroke was almost twice as high as that in the period beyond the first year, although with a wider predictive interval. Specifically, the OR [95% CI] was 4.08 [2.70–6.15] with a predictive interval of [0.97–17.13] in the ≤1-year group, versus 2.28 [1.84–2.83] with a predictive interval of [1.03–5.05] in the >1-year group.

Several subgroup analyses were also conducted based on diagnostic tools (self-administered vs. clinically administered), country income level (as a proxy for socioeconomic status), WHO region, study design, age, and sex. Among these, a significant association was identified between age and the OR of PSD, indicating that younger patients were more likely to develop depression following a stroke.

**Figure 8:**
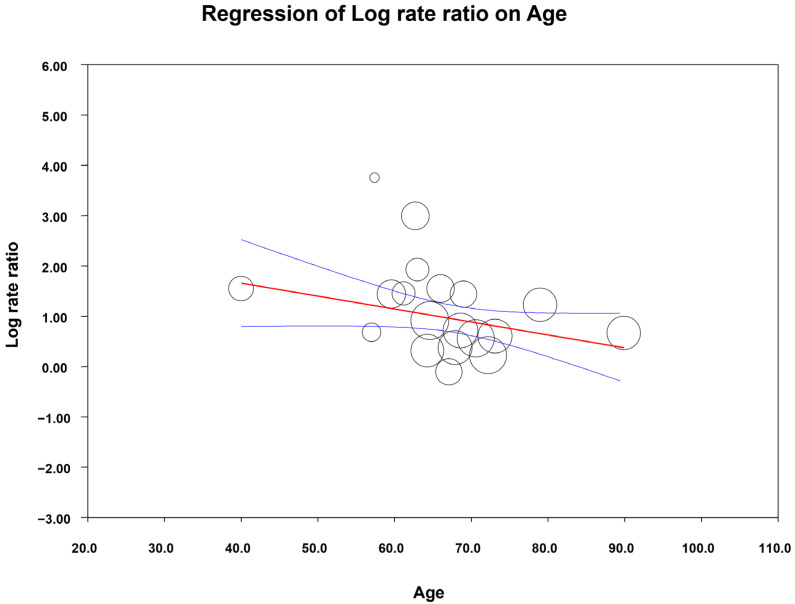
Meta-regression results (age). Circles represent the included primary studies, with their size proportional to its relative weight. The red line is the estimated regression line, while the blue lines indicate the 95% confidence interval for this regression estimate.

A meta-regression analysis was then performed to determine whether mean age predicted the odds of PSD. Age was a significant moderator (β = −0.025, 95% CI: [−0.049 to −0.002]; *p* = 0.030). Although the model only slightly reduced between-study heterogeneity (I^2^ decreased from 90.7% to 87.9%), suggesting that age accounted for a small proportion of variability across studies, it remained a significant predictor of post-stroke depression. The results are reported as a scatter plot in Figure 8.

In the meta-regression model, the studies by Maaijwee et al. [42] and Hornsten et al. [37] appeared as outliers: Maaijwee et al. included only first-stroke patients aged 18–50 years, whereas Hornsten et al. included patients older than 85 years. Notably, removing these two studies did not alter the significant association between age and PSD (β = −0.048, 95% CI: [−0.094 to −0.003]; *p* = 0.035).

It is important to note that 19 out of 27 studies were included in the meta-regression. Therefore, the main meta-analysis was repeated using only these 19 studies, and the results remained virtually unchanged (OR 2.72, 95% CI: [2.28–3.25] compared to the full set of studies (OR 2.71, 95% CI: [2.29–3.22]).

## 4. Discussion

This study is the first systematic review and meta-analysis of comparative studies using OR as the effect size, covering publications up to 23 September 2024. It advances the literature by enabling a more precise evaluation of the individual risk of depression among stroke survivors.

Several meta-analyses of varying quality have been published on the proportional prevalence of PSD. However, these studies typically report depression prevalence in their study populations as percentages, without accounting for the already high prevalence of depression in the general, apparently healthy population, a prevalence that may not be attributable to stroke.

Considering the progressively rising global prevalence of depression, attributing depression solely to the stroke event requires the inclusion of a control group and a focus on OR evaluation, an approach that distinguishes the present study [48].

### 4.1. Result Analysis

#### 4.1.1. OR and Frequency

In recent years, the OR has been increasingly used in the medical literature because it is simple to interpret, easy to calculate, and highly relevant for clinical decision-making. From a clinical perspective, it is valuable for healthcare providers to understand, for example, the odds of treatment success or the likelihood of developing a specific complication.

Moreover, the ease of understanding the OR also makes it a useful tool in communicating with patients, particularly when addressing neuropsychiatric sequelae of acute, debilitating conditions such as stroke [49].

Through this study, we identified a highly concerning finding: post-stroke patients are nearly three times more likely to develop depression compared to the general population (OR = 2.71 [2.29–3.22]). This result is crucial for raising awareness among healthcare providers about the psychological vulnerability of stroke patients, as well as for improving patient compliance with necessary psychiatric treatments or interventions.

Beyond reporting a pooled OR as the novel aspect of our study, we also utilized the search results to provide a pooled estimate of the proportional prevalence of depression among stroke survivors. This analysis was based on 245 PubMed, Web of Science, and SCOPUS reports, including a total of 493,681 patients.

In a recent meta-analysis published by Patra A. et al. in 2021 [50], the authors reported a pooled prevalence of 55%, 95% CI: [42–64] of depression in the Indian population. This figure is higher than the overall prevalence of 32.15% observed in our study, but is consistent with another study conducted in India in 2013 (46.8%). These findings reinforce the hypothesis that genetic or racial variability may influence the prevalence of PSD [39,50].

Similarly, a meta-analysis conducted in Iran by Dalvand S. et al. in 2018 reported an overall prevalence of 46.94%, 95% CI: [30.14–63.75] for PSD, with regional variability ranging from 18% to 72.5% [51]. These results are in line with the findings of our study.

#### 4.1.2. Heterogeneity

In this meta-analysis, we encountered high heterogeneity, which was primarily due to the inherent nature of the included studies. Notably, heterogeneity did not decrease across various subgroup analyses. This outcome is unsurprising, given that the included studies were not matched and reported data from diverse populations across different races, countries, and age groups, using non-uniform depression assessment scales. Nevertheless, this does not imply that conducting a meta-analysis is unfeasible.

A critical review of PSD concluded that small sample sizes are one of the main limitations in the published literature. Regardless of the underlying cause, one effective solution is to design systematic reviews and meta-analyses that pool such small studies to generate more robust and meaningful results [52].

It is nearly impossible to achieve this without accepting a degree of heterogeneity, especially in health science research. As recommended in such cases, applying a random-effects model is the appropriate approach in meta-analyses with substantial heterogeneity [53]. Another useful parameter for interpreting results under these conditions is the predictive interval, which reflects the expected variability of the true effect size in future studies while accounting for observed heterogeneity [54].

**Figure 9:**
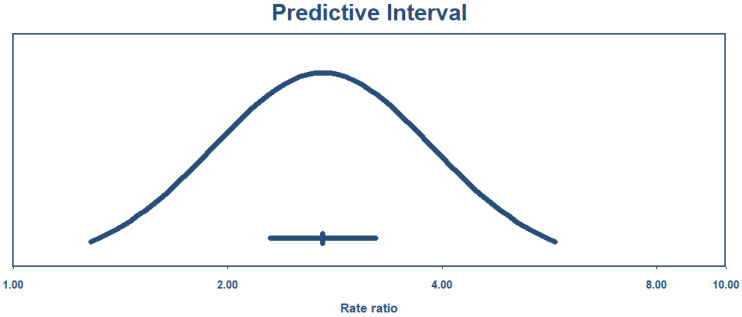
Predictive interval.

As shown in Figure 9, the true effect size with a 95% predictive interval ranges from 1.28 to 5.75, meaning that future studies will report an OR of at least 1.28 with 95% certainty.

#### 4.1.3. Publication Bias

Regarding publication bias, it is important to note that asymmetries in funnel plots do not always indicate the presence of publication bias. This is especially true in the present study, where the results do not show conflicting directions but rather varying effect sizes in the same direction. In fact, the funnel plot reflects “small-study effects”, which may be strongly influenced by heterogeneity rather than publication bias [55].

Considering all the above-mentioned data and the results of the trim-and-fill test, it can be concluded that the findings of the present meta-analysis are not significantly affected by publication bias.

### 4.2. Subgroup Analysis

The natural history of depression in stroke survivors has always been a major concern for clinicians and researchers, and numerous studies with varying designs have been published on this topic. For example, Liu L. et al. [8] recently published a comprehensive systematic review showing that most depression cases have an early onset (within the first 3 months). Moreover, more than half of these early-onset cases are at risk of persistent depression, highlighting the importance of early diagnosis and treatment in the stroke survivor population [8].

In addition to this, Ayerbe L. et al. [56] investigated the natural history of depression in the South London Stroke Register with up to 15 years of follow-up. Their results suggest that most depressive episodes begin within the first year, with one-third of cases diagnosed within the first 3 months, and no new cases reported after year 10. These findings emphasize the dynamic course of PSD: while most stroke survivors experience short-duration episodes, the risk of recurrence remains high in the long term [56].

Overall, nearly all comprehensive studies and reviews are consistent in describing the natural history of PSD. They consider depression an early-onset consequence of stroke, with peak incidence occurring between 6 months and 2 years. It is also evident that depression may persist for several years, with the incidence of new cases declining over time [57].

In our systematic review, we conducted subgroup analyses based on follow-up duration, diagnostic tools (self-administered vs. clinician-administered), country income level (as a proxy for socioeconomic status), WHO region, study design, age, and sex. Although these analyses did not substantially reduce heterogeneity within groups, the results provide valuable insights.

The subgroup analysis by follow-up duration showed that the overall pooled effect and its predictive interval more closely resembled those of studies with follow-up periods longer than one year. Interestingly, the predictive intervals of both the overall effect and the >1-year follow-up group were entirely encompassed by that of the ≤1-year group. These findings suggest that evaluating depression after the first year may yield more stable and persistent estimates compared with assessments conducted within the first year post-stroke, possibly due to greater confounding influences during the acute and subacute phases. It is also important to note that in the >1-year follow-up group, patients were not under continuous treatment or monitoring for depression; rather, the follow-up period was defined as the time point at which depressive symptoms were first assessed after stroke.

Meta-regression analysis performed based on mean age reported in primary studies demonstrated that younger age was a significant risk factor for post-stroke depression (PSD), whereas sex was not significantly associated with PSD risk.

### 4.3. Other Studies

#### 4.3.1. Meta-Analysis

In a meta-analysis conducted by Ayerbe L. et al. in 2013 [58], the authors included 50 studies with percentage as the effect size and reported a pooled prevalence of 29%, 95% CI: [25–32] for depression in patients with a history of stroke, which is very similar to our findings. In the same study, the authors concluded that the principal predictors of PSD are disability, cognitive impairment, pre-stroke depression, anxiety, and stroke severity. They also reported that PSD independently contributes to lower quality of life, increased mortality, and greater disability [58].

One of the most frequently studied and discussed predictors of PSD is pre-stroke depression. In this regard, Taylor-Rowan M. et al. [59], in a meta-analysis with rigorous pre-registered methodology, reported that the pooled prevalence of pre-stroke depression is approximately 12%. Compared to the 39–52% prevalence of PSD [58], this suggests that the majority of PSD cases cannot be explained merely as unmasking or recurrence of pre-stroke depression. According to the authors, most cases of PSD are direct consequences of stroke itself. They further reported that the odds of developing PSD are three times higher in patients with pre-stroke depression compared to those without (OR = 3.0, 95% CI: [2.3–4.0]) [59].

Similarly, Hackett M.L. et al., in their 2014 meta-analysis of 61 primary studies, reported a pooled prevalence of PSD of 31%, 95% CI: [28–35], which was not significantly different from their earlier report in 2005 (33%, 95% CI: [29–36]) [60,61]. This prevalence is almost identical to the results of our study.

Finally, it is important to highlight the association between PSD and mortality. In a study conducted by Bartoli F. et al., it was demonstrated that stroke survivors with depression are at a significantly higher risk of mortality compared to those without depression (RR = 1.50, 95% CI: [1.28–1.75]) [9].

#### 4.3.2. Original Papers

Most of the included studies were conducted on patients with different types of stroke, and the primary papers excluded other possible vascular pathologies that might mimic stroke symptoms, except for the cohort study by Maaijwee, N. A. et al. [42], which included both transient ischemic attack (TIA) and stroke patients. Fortunately, the authors reported the results for two groups separately. In the present meta-analysis, only the data related to stroke patients were used. Interestingly, according to the authors, although both TIA and stroke groups experienced higher rates of depressive symptoms, the OR was higher in ischemic patients compared to TIA patients (4.7 [2.0–22.0] vs. 2.8 [1.2–6.6], respectively) [56].

In a study conducted in China, Zeng, Y. Y. et al. (2021) [62] compared the prevalence of PSD in hemorrhagic stroke survivors and acute ischemic stroke survivors. They concluded that depression is significantly more common among hemorrhagic stroke survivors than among ischemic stroke survivors (42.3% vs. 22.9). After adjusting for confounding variables, the authors reported that the odds of developing depression were more than twice as high in hemorrhagic stroke patients (OR 2.65 [1.34–5.24]) [62].

Several predictors of PSD have been suggested in the literature, including older age, female sex, stroke severity and outcomes, a history of depression or other psychiatric disorders, stressful life events prior to stroke, and lesion location and size [63]. Recent research suggests that the pathophysiology of PSD extends beyond psychosocial factors and is rooted in shared biological mechanisms between stroke and depression. These include chronic neuroinflammation, dysregulation of monoaminergic neurotransmitter systems, and sustained activation of the hypothalamic–pituitary–adrenal (HPA) axis, all of which have been implicated in both conditions [64,65,66]. Elevated inflammatory cytokines, such as IL-6 and TNF-α, and disrupted serotonergic signaling contribute to neuronal apoptosis and impaired neuroplasticity. Neuroanatomical studies further support these findings, highlighting associations between depressive symptoms and lesions within the frontal lobes and basal ganglia, regions involved in emotional processing and executive function [67,68]. Together, these molecular and structural insights underscore the multifactorial etiology of PSD and support the need for early biological and psychological interventions tailored to stroke survivors.

## 5. Limitations and Strengths

This meta-analysis has some limitations. The included studies were highly heterogeneous in terms of design, populations, follow-up duration, and depression assessment tools, and this variability persisted despite subgroup analyses. Furthermore, we only analyzed published studies, excluding unpublished literature, which may have introduced publication bias. In addition, some primary studies were not specifically designed to assess post-stroke depression, and differences in adjustment for confounding factors such as pre-stroke psychiatric history or stroke severity may have influenced the results.

Despite these limitations, the study also has important strengths. Our comprehensive and highly sensitive search strategy initially identified 6088 primary studies, ensuring broad coverage of the literature. The novelty of focusing on comparative studies with OR, together with transparent reporting of the search process, objectives, and inclusion criteria, enhances the reproducibility of our findings. These features allowed us to move beyond simple prevalence estimates and provide a more robust measure of the risk of depression following stroke.

## 6. Conclusions

This meta-analysis aimed to determine the prevalence and odds of depression in stroke survivors compared with a control population. Overall, this systematic review and meta-analysis provides a clearer understanding of depression as a serious and debilitating consequence of stroke. The pooled odds ratio indicates that stroke survivors have nearly three times higher risk of developing depression compared with the general population. This finding underscores the need for systematic and routine screening for depressive symptoms as an integral part of post-stroke care and rehabilitation programs.

Early identification and appropriate treatment of depression in stroke survivors may reduce the risk of persistent symptoms, prevent associated comorbidities, and lower depression-related mortality. Identifying high-risk patients—particularly younger individuals—could facilitate timely psychological or pharmacological interventions, ultimately improving functional recovery, treatment adherence, and quality of life.

Future research should focus on incorporating standardized depression screening tools into stroke care pathways and evaluating whether targeted, risk-based interventions can effectively reduce the incidence and impact of PSD.

## Figures and Tables

**Table 1:** GRADE framework for evaluating evidence certainty on post-stroke depression.

Downgrade criteria	**Domain**	**General Principles in GRADE**	**Criteria for Judgment in Our Study**
	Risk of bias	Confidence in the recommendations decreases when the included studies present important methodological flaws that may distort the estimated associations. Flaws such as inadequate control of confounding factors, inconsistent outcome assessment or lack of transparency in analytical procedures can compromise the validity of results.	In the present study, we evaluated the risk of bias using the Newcastle-Ottawa Scale (NOS), as all included studies were observational in design. All studies demonstrated acceptable methodological quality in relation to our research question. The certainty of the evidence was downgraded when the NOS score was ≤5.
	Inconsistency	Substantial differences in the magnitude or direction of results are referred to as inconsistency. When point estimates diverge markedly or confidence intervals show minimal overlap, the certainty of the evidence may be downgraded. Such variability often reflects unexpected differences in study populations, methodological approaches, or other contextual factors that influence the observed effects.	Inconsistency was explored by examining the variability of individual effect estimates. As a complementary approach, odds ratios lying beyond two standard deviations from the overall mean were considered potential outliers. This threshold (±2 SD) was used to identify studies contributing disproportionately to heterogeneity in the pooled analysis.
	Indirectness	Evidence is considered indirect when the research question of primary studies does not fully correspond to the specific question being evaluated. Indirectness may result from differences in population characteristics, exposure definitions, or outcome measures, thereby limiting the applicability of findings.	Studies that were not primarily designed to assess depression or psychological outcomes after stroke were considered indirect. In such cases, the original study objectives did not fully align with the research question, which may reduce the precision or depth of outcome assessment relevant to this analysis.
	Imprecision	Imprecision arises when estimated effects are surrounded by considerable uncertainty, often reflected in wide confidence intervals. This may result from small sample sizes, few outcome events, or marked variability between study groups, which limit the ability to draw firm conclusions about the true effect.	Studies were considered imprecise when the ratio between the upper and lower limits of the 95% confidence interval exceeded three. In such cases, the certainty of the evidence was downgraded by one level due to the wide uncertainty surrounding the estimated effect.
	Publication bias	The certainty of the evidence may decrease if relevant studies remain unpublished or if results are selectively reported, particularly when studies with null or small effects are less likely to be available. Suspicion of such bias increases when only few studies are published and most are commercially funded.	Publication bias was considered possible when the available evidence suggested selective reporting or incomplete dissemination of results. Studies funded by commercial entities or presenting exclusively positive findings were judged as more prone to this limitation.
Upgrade criteria	Large magnitude of effect	A large or very large effect size observed consistently across studies can increase the certainty of the evidence. When the estimated association is strong and unlikely to be fully explained by residual bias or confounding, confidence in the observed relationship may be upgraded.	Studies showing an odds ratio greater than 2 were upgraded by one level, as such effect sizes indicate a large and consistent association unlikely to be fully explained by residual bias or confounding.
	Plausible confounding	The certainty of the evidence may increase when all plausible sources of bias or unmeasured confounding would reduce, rather than increase, the observed effect. In such cases, the true association is likely to be at least as strong as that reported.	Studies that explicitly adjusted the odds of depression between case and control groups were upgraded by one level.
	Dose-response gradient	A consistent trend showing greater effect with increasing exposure or intensity of the intervention strengthens causal inference. The presence of such a dose–response relationship supports upgrading the quality of the evidence.	Studies that explicitly addressed the association between the severity of depression and stroke severity were upgraded by one level.

**Table 2:** Data extraction table.

First Author’s Name	Publication Year	Stroke Patients’ Age	Sex (Male%)	Sample Size	Depression Prevalence (Among Stroke Patients)	Follow-Up Duration	Depresion Assesment Scale	Study Type	Country	Odds Ratio [95% CI]
Andersen, G. et al. [32].	1994	69	55%	Stroke: 211 Control: 122	25.1%	1 year	HDRS	Prospective case- control	Denmark	4.21 [1.99–8.89]
Beekman, A. T. et al. [33]	1998	N/A	N/A	Stroke: 173 Control: 1026	27.2%	10 years	CES-D	Longitudinal	Netherlands	3.88 [2.60–5.78]
Dam, H. [31]	2001	57 ± 8.5	65.7%	Stroke: 99 Control: 28	19.2%	7 years	HDRS, BDI	Cohort	Denmark	1.97 [0.54–7.24]
Brodaty, H. et al. 3m [34]	2007	N/A	N/A	Stroke: 158 Control: 100	12.0%	3 months	DSM-IV	Longitudinal	Australia	4.15 [1.19–14.41]
Brodaty, H. et al. 15m [34]	2007	N/A	N/A	Stroke: 140 Control: 100	20.7%	15 months	DSM-IV	Longitudinal	Australia	7.23 [2.13–24.54]
Lindén, T. et al. [35]	2007	79 ± 5.3	35%	Stroke: 149 Control: 745	33.6%	20 months	DSM III-R	Case-control	Sweden	3.40 [2.30–5.00]
Fatoye, F. O. et al. [36]	2009	59.6 ± 10.5	57.6%	Stroke: 118 Control: 118	39.6%	11 months	BDI	Case-control	Nigeria	4.22 [2.21–8.02]
Hornsten, C. et al. [37]	2012	89.9 ± 4.5	30.2%	Stroke: 129 Control: 472	50.4%	N/A	GDS-15, MADRS, OBS	Cross- sectional	Sweden	1.94 [1.31–2.88]
Fuller-Thomson, E. et al. [38]	2012	N/A	N/A	Stroke: 858 Control: 65,855	7.4%	N/A	CIDI-SF	Cross- sectional	Canada	2.21 [1.61–3.04]
Jones, M. P. et al. [30]	2012	67.1	28%	Stroke: 51 Control: 58	51%	3 years	HADS	Longitudinal	Tanzania	0.90 [0.42–1.92]
Paul, N. et al. [39]	2013	62.7 ± 13.04	49%	Stroke: 241 Control: 262	46.8%	8–10 months (average 10.79)	bGDS	Case-control within a prospective cohort	India	19.95 [10.09–39.47]
Steptoe, A. et al. [23]	2013	73.1 ± 7.9	53.5%	Stroke: 127 Control: 4852	37.8%	7.8 years	CESD-8	Cohort	England	1.83 [1.27–2.64]
Börsch-Supan, A. et al. [24]	2013	70.6 ± 9.3	55.4%	Stroke: 1082 Control: 25,485	39.6%	3.8 years	EURO-D	Cohort	Europe	1.75 [1.55–1.99]
Mbelesso, P. et al. [40]	2014	N/A	57.1%	Stroke: 35 Control: 70	88.6%	N/A	MADRS	Cross-sectional case-control	Central Africa Republic	19.37 [6.05–62.00]
Sonnega, A. et al. [22]	2014	72.2 ± 10.5	49.6%	Stroke: 820 Control: 12,991	31.6%	6.9 years	CESD-8	Cohort	USA	1.25 [1.07–1.45]
Zhao, Y. et al. [25]	2014	64.3 ± 8.4	56.2%	Stroke: 89 Control: 4932	48.3%	6.9 years	CESD-10	Cohort	China	1.37 [0.90–2.09]
Bulloch, A.G.M. et al. [41]	2015	66	51.6%	Not Specified	22.7%	N/A	CIDI-SFMD	Cross-sectional	Canada	4.70 [2.40–9.40]
Jørgensen, T. S. et al. [10]	2016	N/A	N/A	Stroke: 135,417 Control: 145,499	25.4%	2 years	ICD-10	Cohort	Denmark	4.02 [3.93–4.11]
Maaijwee, N. A. et al. [42]	2016	40	N/A	Stroke: 325 Control: 147	19.5%	10 years	HADS	Cohort	Netherlands	4.70 [2.00–11.00]
Wong, R. et al. [27]	2017	67.9 ± 9.1	49.6%	Stroke: 135 Control: 6855	35.5%	6 years	CESD-9	Cohort	Mexico	1.45 [1.01–2.07]
Oni, O. D. et al. [43]	2018	57.4 ± 9.67	54.3%	Stroke: 70 Control: 70	22.9%	N/A	ICD-10	Cross-sectional case-control	Nigeria	42.68 [2.50–727.47]
Shin, C. et al. [26]	2019	68.6 ± 8.8	56.1%	Stroke: 157 Control: 4625	57.3%	7.9 years	CESD-10A & B	Cohort	Korea	2.04 [1.48–2.82]
Khedr, E. M. et al. [44]	2020	61.2 ± 14.7	60.2%	Stroke: 103 Control: 50	36.9%	N/A	DSM IV TR	Cross-sectional	Egypt	4.28 [1.67–10.99]
Li, X. Y. et al. [29]	2020	N/A	N/A	Stroke: 374 Control: 18,784	6.9%	N/A	WMH-CIDI	Cross-sectional	Canada	1.48 [0.99–2.23]
Lee, E. J. et al. [45]	2022	N/A	53.7%	Stroke: 343 Control: 10,779	21.8%	N/A	PHQ9	Cross-sectional	Korea	2.72 [2.08–3.54]
Choi, H. L. et al. [46]	2023	64.6 ± 12.11	62.4%	Stroke: 207,678 Control: 294,506	33.6%	2 years	ICD-10	Retrospective cohort	Korea	2.49 [2.46–2.53]
Dymm, B. et al. [47]	2024	63 ± 12	51.6%	Stroke: 161 Control: 79	31.7%	2 years	PHQ8	Retrospective analysis of a prospective cohort study	USA	6.86 [2.61–18.00]

Highlighted in red: OR that are not statistically significant.

**Table 3:** Results of the Newcastle–Ottawa Scale risk of bias assessment and GRADE approach for assessing the certainty of evidence. Black stars represent points awarded in each domain of the scale, while white stars indicate points not awarded. Stars in parentheses refer to a single item within the corresponding domain that may contribute up to two points. ⊕OOO = very low evidence quality; ⊕⊕OO = low evidence quality; ⊕⊕⊕O = moderate evidence quality; ⊕⊕⊕⊕ = high evidence quality.

Study Name	Selection	Comparability	Outcome	NOS Score (Risk of Bias Category)	Certainty of Evidence (GRADE)
Andersen, G. et al. (1994) [32]	★★★✰	★✰	★★★	7 (low)	⊕⊕◯◯
Beekman, A. T. et al. (1998) [33]	★★✰✰	★★	★★★	7 (low)	⊕⊕⊕◯
Dam, H. (2001) [31]	★★★★	★★	★★★	9 (low)	⊕◯◯◯
Brodaty, H, et al. (2007) [34]	★★★✰	★★	★★★	8 (low)	⊕⊕◯◯
Lindén, T. et al. (2007) [35]	★★★✰	★★	★★★	8 (low)	⊕⊕⊕◯
Fatoye, F. O. et al. (2009) [36]	★★✰✰	★★	★★✰	6 (average)	⊕⊕◯◯
Hornsten, C. et al. (2012) [37]	★✰★(★★)	★	(★★)★	8 (low)	⊕⊕⊕◯
Fuller-Thomson, E. et al. (2012) [38]	★★★(★✰)	★	(★★)★	8 (low)	⊕⊕⊕⊕
Jones, M. P. et al. (2012) [30]	★★★✰	★★	★★★	8 (low)	⊕◯◯◯
Paul. N. et al. (2013) [39]	★★★★	★★	★★★	9 (low)	⊕⊕◯◯
Steptoe, A. et al. (2013) [23]	★★★✰	✰✰	★★★	6 (average)	⊕⊕◯◯
Börsch_supan, A. et al. (2013) [24]	★★★✰	✰✰	★★★	6 (average)	⊕⊕◯◯
Mbelesso, P. et al. (2014) [40]	★✰✰(★★)	✰	(★★)✰	5 (average)	⊕◯◯◯
Sonnega, A. et al. (2014) [22]	★★★✰	✰✰	★★★	6 (average)	⊕⊕◯◯
Zhao, Y. et al. (2014) [25]	★★★✰	✰✰	★★★	6 (average)	⊕⊕◯◯
Bulloch, A. G. M. et al. (2015) [41]	★★★(✰✰)	★	(★★)★	7 (low)	⊕⊕⊕◯
Jørgensen T. S. et al. (2016) [10]	★★★★	★★	★★★	9 (low)	⊕⊕⊕⊕
Maaijwee, N. A. et al. (2016) [42]	★★★✰	★★	★★★	8 (low)	⊕⊕⊕◯
Wong, R. et al. (2017) [27]	★★★✰	✰✰	★★★	6 (average)	⊕⊕◯◯
Oni, O. D. et al. (2018) [43]	★★★(★★)	✰	(★★)★	8 (low)	⊕⊕◯◯
Shin, C. et al. (2019) [26]	★★★✰	✰✰	★★★	6 (average)	⊕⊕⊕◯
Khedr, E. M. et al. (2020) [44]	★✰✰(★★)	✰	(★★)★	6 (average)	⊕⊕◯◯
Li, X. Y. et al. (2020) [29]	★★✰(★✰)	★	(★★)★	7 (low)	⊕⊕⊕◯
Lee, E. J. et al. (2022) [45]	★★★(★★)	✰	(★★)★	8 (low)	⊕⊕⊕◯
Choi, H. L. et al. (2023) [46]	★★★★	★★	★★✰	8 (low)	⊕⊕⊕⊕
Dymm, B. et al. (2024) [47]	★★★✰	✰✰	★★★	6 (average)	⊕⊕⊕◯

## Supplementary Materials

The following supporting information can be downloaded at: https://www.mdpi.com/article/10.3390/jcm14238410/s1.
