# Supplementary material for: Stroke Survivors Have Almost Three Times Higher Risk of Depression: A Systematic Review and Meta-Analysis
Source: J Clin Med. 2025 Nov 27;14(23):8410. doi: 10.3390/jcm14238410 (PMC12693352; doi:10.3390/jcm14238410)
Supplement: Supplementary file 1 [file jcm-14-08410-s001.zip › jcm-3943783-supplementary.pdf]

ANNEX

NEWCASTLE - OTTAWA QUALITY ASSESSMENT SCALE (adapted for cross sectional studies)

Selection: (maximum 5 stars)

1. Representativeness of the cases:

   a) Truly representative of the HCC patients (consecutive or random sampling of cases).

   1 score

   b) Somewhat representative of the average in the HCC patients (non-random sampling).

   1 score

   c) Selected demographic group of users. 0 score

   d) No description of the sampling strategy. 0 score

2. Sample size:

   a) Justified and satisfactory (≥ 400 HCC included). 1 score

   b) Not justified (<400 HCC patients included). 0 score

3. Non-Response rate

   a) The response rate is satisfactory (≥95%). 1 score

   b) The response rate is unsatisfactory (<95%), or no description. 0 score

4. Ascertainment of the screening/surveillance tool:

   a) Validated screening/surveillance tool. 2 scores

b) Non-validated screening/surveillance tool, but the tool is available or described. 1 score

c) No description of the measurement tool. 0 score

Comparability: (Maximum 1 stars)

1. The potential confounders were investigated by subgroup analysis or multivariable analysis.

a) The study investigates potential confounders. 1 score

b) The study does not investigate potential confounders. 0 score

Outcome: (maximum 3 stars)

1. Assessment of the outcome:

a) Independent blind assessment. 2 scores

b) Record linkage. 2 scores

c) Self-report. 1 score

d) No description. 0 score

2. Statistical test:

a) The statistical test used to analyze the data is clearly described and appropriate. 1 score

b) The statistical test is not appropriate, not described or incomplete. 0 score
